# Counterbalance: modulation of VEGF/VEGFR activities by TNFSF15

**DOI:** 10.1038/s41392-018-0023-8

**Published:** 2018-08-10

**Authors:** Gui-Li Yang, Lu-Yuan Li

**Affiliations:** 1 Tianjin Neurological Institute, Key Laboratory of Head Trauma Clinics and Neuro-Regeneration, Ministry of Education and Tianjin Municipality, 300052 Tianjin, China; 2 State Key Laboratory of Medicinal Chemical Biology, College of Pharmacy, and Collaborative Research Center for Biotherapy, Nankai University, Tianjin, 300071 China

## Abstract

Vascular hyperpermeability occurs in angiogenesis and several pathobiological conditions, producing elevated interstitial fluid pressure and lymphangiogenesis. How these closely related events are modulated is a fundamentally important question regarding the maintenance of vascular homeostasis and treatment of disease conditions such as cancer, stroke, and myocardial infarction. Signals mediated by vascular endothelial growth factor receptors, noticeably VEGFR-1, −2, and −3, are centrally involved in the promotion of both blood vessel and lymphatic vessel growth. These signaling pathways are counterbalanced or, in the case of VEGFR3, augmented by signals induced by tumor necrosis factor superfamily-15 (TNFSF15). TNFSF15 can simultaneously downregulate membrane-bound VEGFR1 and upregulate soluble VEGFR1, thus changing VEGF/VEGFR1 signals from pro-angiogenic to anti-angiogenic. In addition, TNFSF15 inhibits VEGF-induced VEGFR2 phosphorylation, thereby curbing VEGFR2-mediated enhancement of vascular permeability. Third, and perhaps more interestingly, TNFSF15 is capable of stimulating *VEGFR3* gene expression in lymphatic endothelial cells, thus augmenting VEGF-C/D-VEGFR3-facilitated lymphangiogenesis. We discuss the intertwining relationship between the actions of TNFSF15 and VEGF in this review.

## Angiogenesis: Maintaining normal circulation

The ability of tumor necrosis factor superfamily-15 (TNFSF15) protein to balance the actions of vascular endothelial growth factors (VEGFs) highlights new therapeutic strategies for the treatment of diseases that disrupt the circulatory system. Gui-Li Yang at the Tianjin Neurological Institute and Lu-Yuan Li at Nankai University describe the mechanisms through which TNFSF15 inhibits blood vessel growth mediated by VEGF receptor-1 (VEGFR1) and counterbalances the increase in vascular permeability mediated by VEGFR2. Interestingly, TNFSF15 enhances the effects of VEGFR3 on the formation of lymphatic vessels by promoting VEGFR3 gene expression in lymphatic endothelial cells. Further research will determine whether TNFSF15′s unique capacity to regulate the properties of both blood and lymph vessels can be harnessed to improve the treatment of conditions such as cancer, stroke, myocardial infarction and lymphoedema.

## Introduction

Blood vessel walls, except those of capillary vessels, are normally not permeable to blood content such as ions, water, small molecules, proteins, and cells. An increase in vascular permeability is often associated with inflammation under pathological conditions such as wounds, malignant tumors, and cardiovascular diseases.^1–4^ Vascular permeability may be categorized as basal, acute, or chronic.^5,6^ Basal vascular permeability refers to a rapid, intercellular flux of water and salts and, to a limited extent, transcellular passage of plasma proteins across normal capillaries. Acute vascular hyperpermeability refers to the extensive but time-limited passage of plasma and plasma proteins across post-capillary venules by either intercellular or transcellular routes in acute inflammation. Chronic vascular hyperpermeability is the extensive extravasation of plasma and its contents from angiogenic vessels found in cancers, wounds, and sites of chronic inflammation.^5,7^

Signals derived from the actions of the family of vascular endothelial growth factors (VEGFs) and their receptors (VEGFRs) are the main modulators of angiogenesis and vascular permeability. The most well-studied member of this cytokine family is VEGF, originally discovered as vascular permeability factor (VPF).^8,9^ VEGF is markedly upregulated in cancers^2^ and in ischemic conditions such as stroke^10^ and myocardial infarction.^11^ Deletion of the *VEGF* gene in mice results in aborted vascular development and embryonic lethality.^12^ Promotion of microvascular permeability is the most distinctive biologic activity of VEGF.^13^ Upregulation of VEGF leads to the formation of a porous vasculature that contributes to edema during the acute stage of cerebral stroke^14^ and persistent inflammation in cancers.^15^ Abnormal vascular permeability also impedes the penetration of anticancer drugs because of high interstitial fluid pressure in tumors.^16,17^ Increased interstitial fluid pressure is attributed significantly to lymphatic vessel growth.^18^ Two members of the VEGF family of genes, VEGF-C/D, and their receptor VEGFR3, which is expressed considerably more frequently in lymphatic endothelial cells than in other types of cells,^19,20^ play a critical role in promoting lymphangiogenesis.

Tumor necrosis factor superfamily-15 (TNFSF15) is a cytokine that seems to systematically counterbalance VEGF/VEGFR activities regarding angiogenesis and vascular permeability.^21–23^ TNFSF15 is capable of inhibiting VEGFR1 and VEGFR2 activities on vascular endothelial cells and, quite interestingly, augmenting VEGFR3 activity in lymphatic endothelial cells, thus influencing lymphangiogenesis.^23–25^ Additionally, TNFSF15 can inhibit *VEGF* gene expression,^26^ and the gene expression of TNFSF15 itself can also be downregulated by VEGF.^27^ TNFSF15 and VEGF thus constitute a paired system that provides balanced actions in the modulation of vascular homeostasis. It is highly unique that one cytokine exerts a significant impact on three signaling pathways mediated by three different ligand-receptor systems, albeit the same family of genes. We focus in this review on the intertwining signaling pathways activated by VEGFs and TNFSF15 in the modulation of vascular stability, angiogenesis, and lymphangiogenesis.

## THE VEGF-INDUCED SIGNALING PATHWAY LEADS TO ENHANCED VASCULAR PERMEABILITY

### The VEGF family of growth factors and their receptors

The VEGF family of genes includes five members, VEGF-A (VEGF), VEGF-B,^28^ VEGF-C,^29^ VEGF-D,^30^ and placental growth factor (PlGF).^31^ VEGF and its receptors VEGFR1 and VEGFR2 play a crucial role in vascular endothelium development. VEGF interacts with its cell-surface receptors VEGFR1 (also known as Flt-1),^32,33^ VEGFR2 (also known as Flk-1/KDR),^34,35^ and neuropilin-1.^36^ A truncated, soluble form of VEGFR1 (also known as sFlt1) that results from alternative splicing is found in serum and retains VEGF-binding activity; thus, it may compete with VEGFR1 for VEGF and function as an inhibitor of VEGF activity.^37,38^ VEGFR2 appears to be the principal player in the mediation of signals induced by VEGF that leads to increased vascular permeability and endothelial cell proliferation.^39,40^ VEGF binding to VEGFR2 causes conformational changes and dimerization of the latter,^36^ followed by phosphorylation of several tyrosine residues of the receptor molecule^41^ that, in turn, leads to a cascade of signals that cause enhanced permeability of the blood vessel wall.^39^ VEGFR3, of which much is only beginning to be learned, is the receptor for VEGF-C and -D and is important for lymphatic endothelial cell development and function.^25,42–44^ Recently, it was shown that VEGFR3-mediated signals may, under certain experimental conditions, interact with those mediated by VEGFR2 to downmodulate vascular permeability.^45^

### The VEGF-VEGFR2-eNOS signaling pathway

Endothelial nitric oxide synthase (eNOS) is one of the first identified genes activated by VEGF.^46^ Activation of eNOS and production of nitric oxide (NO) play a prominent role in VEGF-induced vascular permeability. Utilizing permeability models and various signaling pathway inhibitors, several signaling pathways, including the eNOS-cGMP pathway,^47^ PLC-gamma-PKC-Ca^2+^-eNOS^48^ and PI3K-PKB/Akt-eNOS pathway,^49^ have been described as essential to VEGF modulation of vascular permeability. For example, Akt, as a key moderator of vascular permeability, enables phosphorylation of the serine residue 1179 of eNOS, directly causing activation of this enzyme and NO production.^50^ A dominant-negative mutant of Akt is able to inhibit VEGF-induced vascular permeability, and a constitutively active form of Akt can stimulate vascular permeability in a manner similar to recombinant VEGF protein administration,^51^ although Akt-mediated increased vascular permeability can still be blocked by the eNOS inhibitor L-NAME.^51^ VEGF-induced permeability is significantly suppressed in both quiescent and angiogenic blood vessels in *eNOS*^*−/−*^ mice compared with those in wild-type or *iNOS*^*−/*^^−^ mice.^52^ These findings indicate that eNOS is pivotal to VEGF modulation of vascular permeability.

### The VEGF-VEGFR2-Src-VE-cadherin signaling pathway

VEGF is able to activate Src kinase in endothelial cells,^36^ and inhibition of Src kinase activity leads to retarded Evans blue extravasation in mice.^53^ Under physiological conditions, a VEGFR2/VE-cadherin/β-catenin complex was shown to exist in quiescent blood vessels, and VEGF stimulation was demonstrated to result in several events, including disassociation of the complex^11^ and phosphorylated Vav2 (a guanine nucleotide-exchange factor) in a Src-dependent manner.^54^ Additionally, Rac activation by VEGF is known to promote p21-activated kinase (PAK)-mediated phosphorylation of a highly conserved motif of VE-cadherin, resulting in the recruitment of β-arrestin2 to serine-phosphorylated VE-cadherin, such that rapid endocytosis of VE-cadherin occurs, concomitant with a disruption of endothelial barrier function.^54^ Moreover, blockade of Src with protein phosphatase-1 (PP1) can prevent disassociation of the VEGFR2/VE-cadherin/β-catenin complex and inhibit VEGF-mediated vascular permeability.^11^ These findings are consistent with the view that Src plays a very important role in promoting vascular permeability.

### The VEGF-VEGFR2-FAK-VE-cadherin signaling pathway

Focal adhesion tyrosine kinase (FAK) is not a receptor or membrane-associated.^55^ Activation of FAK by VEGF, however, is part of an important signaling pathway leading to enhanced vascular permeability because the loss of *FAK* gene expression can render the inability of VEGF to induce vascular permeability.^56^ It has been shown^57^ that VEGF-induced vascular permeability, FAK activation, and regulation of adhesion junction are attenuated in FAK-kinase-dead (FAK-KD) knockin mice. These findings suggest that FAK is a key regulator of the integrity of vascular barrier. Furthermore, the stimulation of VEGF has been shown to trigger a conformational change of FAK from an inactive to an active form,^57^ which then interacts with VE-cadherin and catalyzes the phosphorylation of β-catenin, which is associated with VE-cadherin. This process, in turn, leads to dissociation of β-catenin and α-catenin from VE-cadherin, resulting in the collapse of endothelial cell junctions.

### The VEGF-VEGFR2-integrin α_v_β_3_ signaling pathway

Integrin ανβ3 is expressed in human wound granulation tissue (angiogenic vascular tissue) but not in normal skin (quiescent vascular tissue),^58^ suggesting that integrin ανβ3 plays a crucial role in angiogenesis. VEGF stimulation can promote the association of integrin ανβ3 with VEGFR2, which is required for VEGFR2 activation.^59–61^ Integrin ανβ3-deficient mice exhibit elevated levels of VEGFR2, enhanced blood vessel growth under pathological conditions,^62,63^ and increased vascular permeability.^64^ Inhibition of VEGFR2 activity using the neutralizing anti-Flk-1 antibody DC101 can eliminate VEGF-induced blood vessel permeability in β3-deficient mice.^64^ These findings indicate that integrin ανβ3-facilitated upregulation of VEGFR2 levels under these experimental conditions is responsible for VEGF-stimulated blood vessel permeability. Additionally, because integrins are also critical parts of the architecture of the cytoskeleton, blockade of integrin αvβ3 in mice can enhance vascular leakage, apparently because of the inhibition of endothelial cortical actin formation,^65^ attributable to the side effect of blood vessel leakage when integrin αvβ3 activity is blocked. Moreover, because integrin interactions with matrix proteins such as fibronectin and vitronectin are important to the maintenance of endothelial barrier function, inhibition of integrin binding to these matrix proteins can lead to increased vascular permeability.^66^

## MODULATION OF VEGF/VEGFR ACTIVITIES BY TNFSF15

### Biological activities of tumor necrosis factor superfamily-15

TNFSF15, also known as vascular endothelial cell growth inhibitor (VEGI) or TL1A, is a cytokine produced largely by vascular endothelial cells.^67–69^ Various activities have been attributed to this unique cytokine, the first one being that it specifically inhibits endothelial cell proliferation by inducing apoptosis in proliferating endothelial cells in angiotensin blood vessels, but not in quiescent endothelial cells in established blood vessels.^68,70–72^ The intracellular signals involved in TNFSF15-stimulated endothelial cell apoptosis include the inactivation of Akt and activation of stress-activated protein kinase/c-Jun N-terminal protein kinases (SAPKs/JNKs), p38 mitogen-activated protein kinase (p38 MAPK) and caspase-3.^72,73^ The ability of TNFSF15 to inhibit angiogenesis apparently can result in the inhibition of tumor growth in experimental animals.^69^

TNFSF15 is also able to inhibit the differentiation of bone marrow-derived endothelial progenitor cells (EPCs) into endothelial cells and the incorporation of EPC into the tumor neovasculature.^74,75^ Interestingly, TNFSF15 does not induce apoptosis in early-stage EPC, unless these cells have differentiated into endothelial cells. In early-stage EPC, TNFSF15 treatment leads to Erk phosphorylation; however, in the later stage of EPC, the treatment leads to activation of NF-κB, JNK, and caspase-3.^74^

Interestingly, TNFSF15-induced endothelial cell apoptosis is cell cycle dependent, because TNFSF15 treatment causes the growth arrest of quiescent endothelial cells in G_0_/G_1_, but eradication of the cells once they have entered late G_1_ stage of the growth cycle.^76^ These findings suggest that the main function of this cytokine is the maintenance of vascular homeostasis.

### Death receptor-3 mediates TNFSF15 activities

TNFSF15 has been shown to be able to activate T-cells^67^ and promote dendritic cell maturation.^77^ In these immune cells, TNFSF15 induces NF-κB activation and IκBα degradation.^67,77,78^ When acting on bone marrow-derived immature dendritic cells, TNFSF15 not only activates the IKB/NF-κB pathway but also the MAPK and JAK/STAT pathways, with STAT3, MAPK p38, and JNK activated, while ERK1/2 phosphorylation is downregulated.^77^ Death receptor-3 (DR3) is currently the only known mediator of TNFSF15 actions on immune cells,^67^ lymphatic endothelial cells^25^ and vascular endothelial cells.^73^ TNFSF15-DR3 interaction results in the recruitment of several adaptor proteins, including TRADD, the platform adaptor, signal transducer TRAF2, RIP and c-IAP1, to DR3 to form a signaling complex, thereby triggering downstream signaling pathway.^78^

### TNFSF15 changes the VEGF/VEGFR1 signaling system from pro-angiogenic to anti-angiogenic

**Fig. 1:**
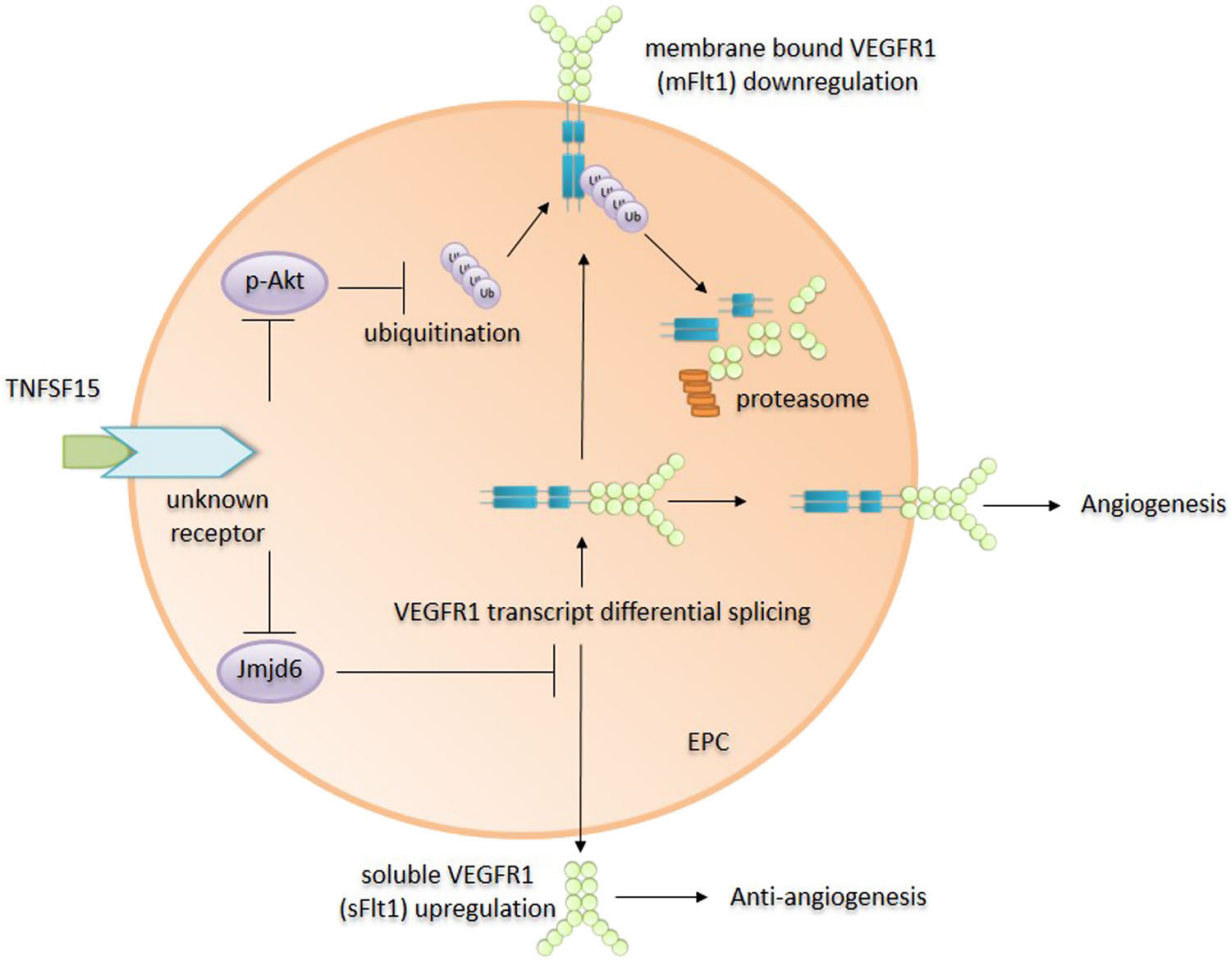
Plausible mechanisms of TNFSF15 actions that change the VEGF-VEGFR1 system from pro-angiogenic to anti-angiogenic. TNFSF15 action on EPC, apparently via a currently unidentified receptor, facilitates Akt deactivation-dependent degradation of membrane-bound, full-length VEGFR1 (mFlt1), which otherwise mediates the angiogenesis-promoting activity of VEGF. Concurrently, TNFSF15 action results in the alleviation of Jmjd6-inhibited production of soluble VEGFR1 (sFlt1), an inhibitor of angiogenesis

VEGFR1 functions are attributed to vascular structural organization,^79,80^ including the promotion of endothelial cell proliferation and migration.^81,82^ While VEGFR1 is probably not the main mediator of VEGF-induced signals regarding vascular permeability, it is responsible for stimulating angiogenesis, which is almost always preceded with the destabilization of blood vessels.^5^ Consistent with an inhibitory role in tumor neovascularization,^74,75^ TNFSF15 has been shown to be capable of suppressing VEGF-facilitated, EPC-supported vasculogenesis by changing VEGF/VEGFR1 activity from pro-angiogenic to anti-angiogenic.^24^ There are two isoforms of VEGFR1: a membrane-bound form (mFlt1), which is the full-length, fully functional receptor for VEGF, and a soluble protein (sFlt1) comprising only the extracellular segment of VEGFR1 that can compete with VEGFR1 for VEGF and act as an inhibitor of VEGF activity.^37^ TNFSF15 treatment of EPC leads to accelerated degradation of mFlt1 and enhanced production of sFlt1, thus inhibiting VEGF-stimulated blood vessel growth in experimental animals.^24^ Mechanistically, it was shown that, while promoting the VEGFR1 gene Flt1 transcription by activating the PKC, Src, and Erk1/2 signaling pathway, TNFSF15 facilitates Akt-deactivation-dependent, ubiquitin-assisted degradation of mFlt1 and, at the same time, promotes alternative splicing of the Flt1 mRNA in favor of sFlt1 by downregulating nuclear protein Jumonji domain-containing protein 6 (Jmjd6), thus alleviating Jmjd6-inhibited sFlt1 expression.^24^ Changing the VEGF/VEGFR1 system from pro-angiogenic to anti-angiogenic may, in part, account for the anti-angiogenic activity of TNFSF15 (Fig. 1).

### TNFSF15 inhibits VEGF-induced vascular hyperpermeability by inhibiting VEGFR2 phosphorylation

**Fig. 2:**
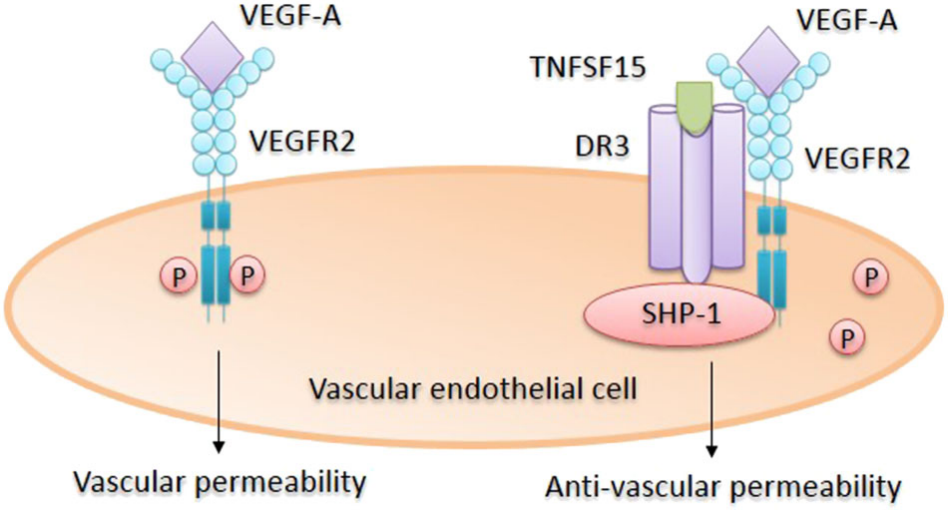
Inhibition of VEGF-stimulated vascular permeability by TNFSF15. TNFSF15 action on endothelial cell leads to the recruitment of phosphatase SHP-1 to the VEGFR2/DR3 protein complex where SHP-1 is able to catalyze VEGFR2 dephosphorylation

VEGF-induced phosphorylation of VEGFR2 is likely the most essential event in VEGFR2-mediated signals, leading to the enhancement of vascular permeability. Several factors are known to downregulate VEGFR2 activity regarding vascular permeability, including TIMP-2,^83,84^ thrombospondin-1,^85,86^ and dopamine.^87,88^ However, that TNFSF15 functions as a negative regulator of VEGFR2 activation may be of particular importance because *TNFSF15* gene expression is prominent in quiescent endothelial cells in established blood vessels in normal tissues but is downregulated in proliferating endothelial cells.^76^ Additionally, TNFSF15 production can be downregulated by VEGF and other proinflammatory factors such as MCP1, in experimental tumor models.^89,90^ In a recent study, VEGF-induced vascular permeability was shown to be inhibited in mice by intraperitoneally administered recombinant human TNFSF15.^23^ In another animal model in which systemic TNFSF15 protein levels were significantly elevated transgenically, the experimental animals became resistant to the VEGF-stimulated increase in vascular permeability.^23^ Inhibition of vascular permeability by TNFSF15 is attributed to the ability of TNFSF15 to inhibit VEGF-induced phosphorylation of a tyrosine residue on the VEGFR2 molecule. Mechanistically, VEGFR2 has been shown to be able to form a protein complex with DR3, the latter being the membrane surface receptor of TNFSF15^73^; in response to TNFSF15 action, a protein tyrosine phosphatase, SHP-1,^91^ is recruited to the protein complex, resulting in the dephosphorylation of VEGFR2. The ability of TNFSF15 to directly induce VEGFR2 deactivation is consistent with the potential role of this cytokine in maintaining vascular homeostasis (Fig. 2).

### Augmentation of VEGF-C/VEGFR3-induced lymphatic endothelial cell growth by TNFSF15

The lymphatic vessels as part of the circulation system regulate interstitial fluid balance in both healthy and disease conditions. Excessive interstitial fluids arising from enhanced vascular permeability may be cleared by lymphangiogenesis and lymphatic circulation. In several pathological settings, increased microvascular permeability to proteins, anaerobic evolution of toxic metabolites and decreased cardiac contractility may cause hypoxic injury and, consequently, increased interstitial colloid pressure and fluid accumulation.^92^ Inadequate lymph drainage may result in lymphedema. Signals derived from the VEGF-C/D-VEGFR3 system are the main forces driving lymphangiogenesis.^19,20^ TNFSF15 was found to promote *VEGFR3* gene expression in lymphatic endothelial cells.^25^ This observation is somewhat surprising because the activities that TNFSF15 exerts on the other two members of the VEGFR family, VEGFR1 and VEGFR2, are principally inhibitory. However, this capability of TNFSF15 is in good agreement with the hypothesis that it is involved in the maintenance of normal circulation.

The lymphatic vessel-promoting activity of TNFSF15 is mediated by DR3, whose activation by TNFSF15 leads to NF-kappa B activation, facilitated *VEGFR3* gene expression and, consequently, expedited lymphatic endothelial cell growth in response to VEGF-C stimulation.^25^ Markedly enhanced lymphangiogenesis and lymph drainage are observed in TNFSF15-overexpressing transgenic mice, which exhibit elevated TNFSF15 levels in the circulation.^25^ More strengthened lymphangiogenesis is also seen in the embryos of transgenic mice.

**Fig. 3:**
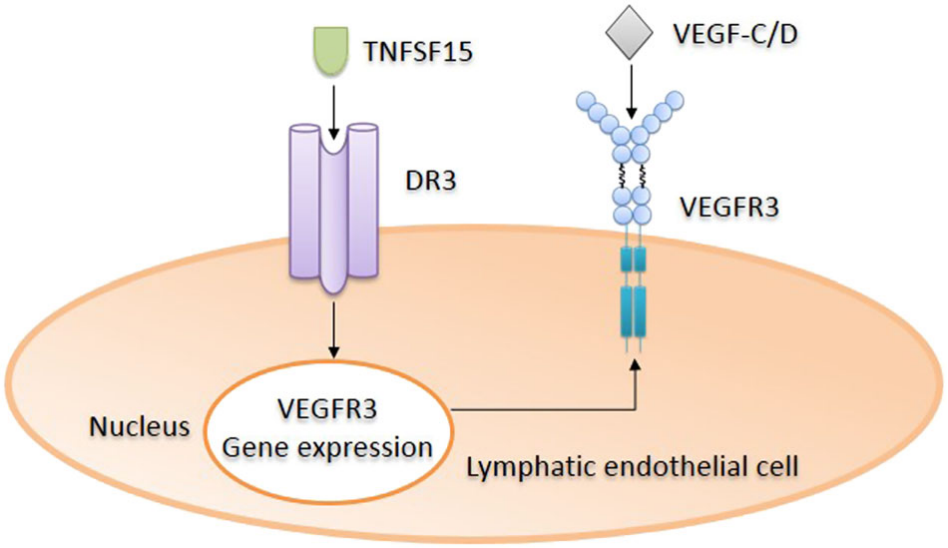
Promotion of *VEGFR3* gene expression by TNFSF15 in lymphatic endothelial cells. TNFSF15 interaction with DR3 in LEC leads to activation of the NF-κB signaling pathway and upregulation of *VEGFR3* gene expression, facilitating VEGF-C/D-driven, VEGFR3-mediated LEC proliferation, migration, and lymphangiogenesis

That TNFSF15 is able to enhance VEGFR3 expression and promote lymphangiogenesis has profound implications clinically. Intervention of lymphoedema may be achieved through TNFSF15-enhancement of the VEGFR3-mediated signaling pathways.^93^ Additionally, it was reported that VEGFR3 has an inhibitory activity on VEGFR2 expression and VEGF/VEGFR2 signaling in vascular endothelial cells, thereby preventing the development of excessive vascular permeability.^45^ Because low levels of VEGFR3 are also present in vascular endothelial cells in a stable vasculature,^94,95^ the upregulation of *VEGFR3* gene expression by TNFSF15 likely contributes to the maintenance of vascular stability (Fig. 3).

### TNFSF15 and VEGF mutually inhibit gene expression

TNFSF15 expression has been reported to be markedly downregulated in inflammatory tissues such as cancer lesions^89^ and wounds.^96^ Downregulation of *TNFSF15* gene expression in cancer tissues in clinical settings was found to be driven by VEGF and MCP-1,^89,90^ suggesting that the downmodulation of TNFSF15 activity is a prerequisite of tumor neovascularization. VEGF is able to stimulate the production of several microRNAs, such as miR-20a and miR-31, which target the 30-UTR of TNFSF15.^27^ Treatment of endothelial cells with the Akt inhibitor LY294002 results in diminished miR-20a and miR-31 production, whereas the Erk inhibitor U0126 prevents VEGF-stimulated expression of miR-20a. Inactivation of either Erk or Akt restores *TNFSF15* gene expression. These findings suggest that VEGF-stimulated production of miR-20a and miR-31 is the probable cause of TNFSF15 downregulation.

On the other hand, *VEGF* gene expression can be suppressed by TNFSF15.^26^ Blocking TNFSF15 activity using either a siRNA against DR3 or a neutralizing antibody against TNFSF15 leads to reinvigoration of VEGF production. The mechanism involves a microRNA, miR-29b, whose targets include the VEGF gene. Because *TNFSF15* gene expression is more prominent in a quiescent vasculature than an angiogenic one, the inhibition of *VEGF* gene expression by TNFSF15 likely occurs in normal tissues where blood vessels are stable, thus contributing to the stability of an established vasculature.

## OUTLOOKS

**Fig. 4:**
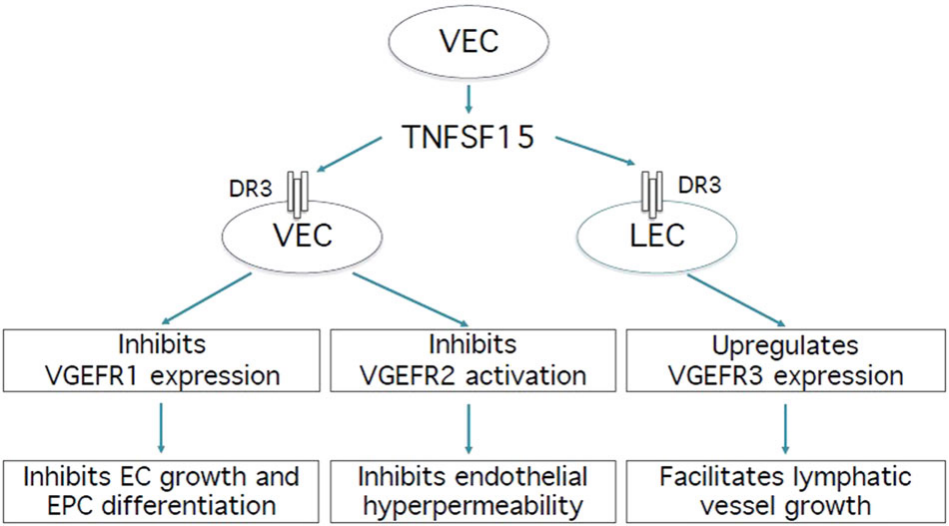
A schematic representation of TNFSF15 modulation of VEGFR activities. TNFSF15 is produced largely by vascular endothelial cells and targets, most noticeably, vascular endothelial cells (VECs) and lymphatic endothelial cells (LECs). TNFSF15 action on VEC leads to inhibition of VEGFR-mediated angiogenesis. TNFAF15 action on VEC also induces dephosphorylation of VEGFR2, thus blocking VEGF-induced, VEGFR2-mediated vascular hyperpermeability. In sharp contrast, TNFSF15 action on LEC leads to elevated VEGFR3 expression, LEC proliferation, and lymphatic vessel growth

Because TNFSF15 can inhibit VEGFR1-mediated vasculogenesis and VEGFR2-mediated vascular hyperpermeability and concurrently promote VEGFR3-mediated lymphangiogenesis, this cytokine may be in a unique position to simultaneously modulate vasculogenesis and lymphangiogenesis (Fig. 4). Vascular hyperpermeability, which often precedes angiogenesis, and lymphangiogenesis are closely linked events regulated by VEGFR2 and VEGFR3, respectively. In mouse embryos, lymphatic endothelial cells differentiate from vascular endothelial cells in the cardinal vein.^97,98^ VEGFR2 and VEGFR3 seem to carry out their functions cooperatively. Although VEGFR3 is considered a lymphatic endothelial cell marker, the expression of VEGFR2 and VEGFR3 can occur concomitantly in endothelial cells.^99^ While VEGFR2 deletion has little effect on developmental lymphangiogenesis, VEGFR3 expression is essential to postnatal lymphangiogenesis^100^ and can facilitate blood vessel sprouting in the presence of VEGFR2.^100^ On the other hand, VEGFR3 expression in blood vessels has an effect of downregulating VEGFR2 expression, thus curbing excessive vascular permeability.^45^

There are several ways that TNFSF15 may be therapeutically useful. The ability to inhibit VEGF/VEGFR1-driven neovascularization makes TNFSF15 a good candidate to treat angiogenesis-facilitated diseases. The ability to block VEGF-induced VEGFR2 activation coupled with the ability to promote lymphatic vessel growth by stimulating *VEGFR3* gene expression in lymphatic endothelial cells may allow TNFSF15 to be used to treat vascular hyperpermeability-related disease conditions. The mutual inhibition of *TNFSF15* and *VEGF* gene expression is also an interesting aspect of the counterbalancing actions of these two cytokines, potentially allowing the use of TNFSF15-induced microRNA molecules, which target *VEGF* gene expression, as therapeutic agents. Furthermore, the lymphatic vasculature plays a pivotal role in shaping immunity, including that in cancers.^18,101,102^ The ability of TNFSF15 to stimulate lymphatic vessel formation warrants further investigation, perhaps especially focusing on its potential application in the enhancement of cancer immunotherapy.
